# Barriers and facilitators for mental healthcare in pediatric lupus and mixed connective tissue disease: a qualitative study of youth and parent perspectives

**DOI:** 10.1186/s12969-015-0049-1

**Published:** 2015-11-24

**Authors:** Andrea M. Knight, Michelle E. Vickery, Alexander G. Fiks, Frances K. Barg

**Affiliations:** Division of Rheumatology, Children’s Hospital of Philadelphia, 3405 Civic Center Blvd, Philadelphia, PA 19104 USA; Center for Pediatric Clinical Effectiveness, Children’s Hospital of Philadelphia, 3535 Market St. 15th Flr, Philadelphia, PA 19104 USA; PolicyLab, Children’s Hospital of Philadelphia, 3535 Market St. 15th Flr, Philadelphia, PA 19104 USA; Division of General Pediatrics, Children’s Hospital of Philadelphia, 3401 Civic Center Blvd, Philadelphia, PA 19104 USA; Mixed Methods Research Lab, University of Pennsylvania, 8th Flr Blockley Hall, 423 Guardian Drive, Philadelphia, PA USA; Department of Family Medicine and Community Health, University of Pennsylvania, 141 Anatomy and Chemistry Bldg, Philadelphia, PA USA; Center for Clinical Epidemiology and Biostatistics, University of Pennsylvania, 815 Blockley Hall, 423 Guardian Drive, Philadelphia, PA USA

**Keywords:** Pediatric rheumatology, Lupus, Mixed connective tissue disease, Depression, Anxiety

## Abstract

**Background:**

Untreated mental health problems may result in poor outcomes for youth with systemic lupus erythematosus (SLE) and mixed connective tissue disease (MCTD). We investigated perceptions, barriers and facilitators for mental healthcare of these youth.

**Methods:**

We conducted 32 semi-structured interviews with 16 outpatient youth with SLE/MCTD, ages 11–22 years, and their parents. We used purposive sampling to deliberately obtain the experiences of youth screened during a previous study for depression and anxiety with the Patient Health Questionnaire 9 and the Screen for Childhood Anxiety and Related Disorders, respectively. We recruited 6 youth with previous positive screens and 10 with negative screens. We assessed interim mental health history, and qualitatively examined perceptions, barriers and facilitators for mental healthcare.

**Results:**

Youth with a mental health history increased from 6 (38 %) at initial screening to 9 (56 %) at interview (mean follow-up = 2.1 years). Youth receiving mental health treatment increased from 33 to 67 %. Youth and parents identified rheumatologists as primary physicians and found mental health screening in rheumatology acceptable. Barriers to mental healthcare included: stigma; fear; uncertainty about getting help; parental emotional burden; minimization by doctors; and limited mental healthcare access. Facilitators included: strong clinician relationships; clinician initiative, sincerity and normalization in discussing mental health; and increased patient/family awareness of mental health issues in SLE/MCTD.

**Conclusion:**

Youth with SLE/MCTD and their parents perceive pediatric rheumatologists as a preferred source for mental health screening, guidance and referral. Interventions addressing barriers and enhancing facilitators may improve mental healthcare for youth with SLE/MCTD.

## Background

Systemic lupus erythematosus (SLE) and the SLE-like syndrome of mixed connective tissue disease (MCTD) are chronic autoimmune conditions associated with significant morbidity and mortality due to multi-organ damage and side effects of long-term, high-risk immunosuppressive treatment [1]. Mental health problems such as depression and anxiety are common in youth with SLE [2–4] and are under-recognized and undertreated [5]. In contrast to the prevalence of depression at 11 % and anxiety at 8 % of the United States general adolescent population [6, 7], these disorders occur in 20–55 % and 20–35 %, respectively, of youth with SLE/MCTD [2, 5, 8]. While the cause remains unclear, potential reasons include CNS inflammation [9], the psychological burden of chronic disease, effects of steroid treatment, social, cultural and genetic factors. Mental health intervention is important because depression and anxiety are associated with poor clinical and psychosocial outcomes in adults with SLE [10–12], as well as youth with other chronic diseases [13–15].

The US Preventive Services Task Force recommends depression screening for adolescents when systems of care are in place to ensure accurate diagnosis and treatment [16]. In primary care, studies indicate that collaborative mental and medical care models effectively connect affected youth to mental health services [17]. However, these systems may miss youth such as those with SLE who primarily rely on subspecialty care and may benefit from mental health intervention in subspecialty settings. In a previous cross-sectional survey study examining the prevalence of depression and anxiety and their association with healthcare utilization in youth with SLE/MCTD, we found that those with depression were less likely to visit their primary care doctor than those without depression, but they were equally as likely to visit their rheumatologist [5]. Rheumatologists may therefore have a central role in intervention for affected youth with SLE/MCTD, but it is unknown whether these youth and their parents would find this acceptable.

In this qualitative analysis we sought to explore patient and parent perspectives on mental healthcare for youth with SLE/MCTD. Qualitative methods use a systematic approach to analyzing empirical information, and are a valuable tool for understanding complex, real-world phenomena such as patient experiences with aspects of healthcare [18]. Purposive sampling, often used in qualitative methods, involves non-random, deliberate sampling from pre-defined groups to maximize collection of relevant data [19]. To gain insight on the mental healthcare experience of youth with SLE/MCTD, we recruited a purposive sample of these youth and their parents who had participated in depression and anxiety screening during our previous study. We aimed to: 1) qualitatively characterize their beliefs about mental health and perceived barriers and facilitators to mental healthcare, and 2) assess rates of interim depression, anxiety and mental health treatment in youth with SLE/MCTD.

## Methods

### Setting

The study was conducted from July 2014 to April 2015 at The Children’s Hospital of Philadelphia (CHOP) Division of Rheumatology, which serves an annual volume of approximately 100 adolescents with SLE/MCTD, and was staffed by 13 physicians, 3 nurses and 1 social worker during the study period.

### Participants

**Fig. 1:**
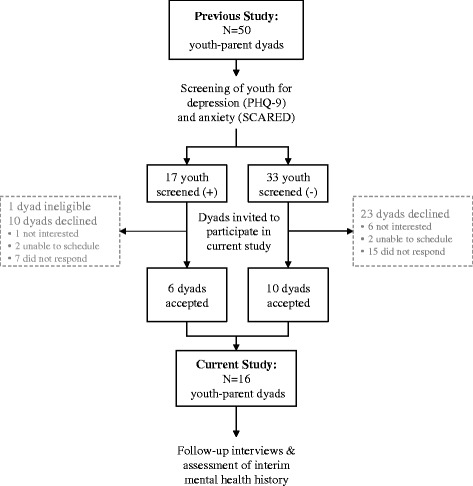
Flow diagram of sampling and participation in the study. Shown is the purposive sampling strategy utilized to recruit youth with SLE/MCTD and their parents for participation in the interview study. Youth-parent dyads who had participated in a previous cross-sectional study, during which youth were screened for depression and anxiety, were invited to participate. Sixteen dyads accepted and underwent interviews and assessment of their interim mental health history. Of the 33 dyads declining to participate, 7 were not interested, 4 expressed interest but were not successfully scheduled for interview (all were followed at CHOP rheumatology but 3 were away at college), and 22 did not respond (7 were no longer followed at CHOP; 8 were followed at CHOP but were away at college). One dyad was deemed ineligible due to co-occurring developmental disorder

We recruited 16 pairs of youth and their parents (or legal guardians). Youth were eligible for inclusion if they: 1) had a diagnosis of SLE (fulfilling ≥4 of 11 SLE classification criteria) [20] or MCTD (fulfilling either Kahn’s or Alarcon-Segovia’s criteria) [21] with pediatric-onset before the 18th birthday; 2) were ages 8 years and above, and; 3) had participated in a previous cross-sectional study of depression and anxiety in pediatric SLE/MCTD [5]. Parents of eligible youth were eligible for inclusion. All 50 youth-parent dyads in the previous study were invited to participate in-person, by phone, mail or email; 16 dyads accepted, 33 declined and one was deemed ineligible due to co-occurring developmental disorder (Fig. 1).

### Data collection and analysis

We conducted a semi-structured individual interview with each of the 16 youth and 16 parents, during which open-ended questions were followed by specific prompts to elicit perceptions, barriers and facilitators for both identification and treatment of mental health problems. An interviewer trained in qualitative techniques and not involved in patient care administered separate interview guides for youth and parents containing the questions and prompts. Interviews took place in-person (*n* = 13) at the CHOP rheumatology clinic or via telephone (*n* = 19). Telephone interviews were offered to maximize availability for participation; they have been shown to yield comparable content to in-person interviews in children [22], and have benefits for increasing perceived subject anonymity during discussion of sensitive health topics [23]. All interviews were digitally recorded, transcribed, de-identified and entered into NVivo software (QSR International Pty Ltd., Version 10, 2014). We conducted thematic analysis of the textual data to identify common themes or patterns across participants pertaining to mental health care. Using an integrated approach we sorted data into broad subject areas by assigning “codes” to specific transcript comments, utilizing both a priori codes that reflected our key research question alongside inductive codes that emerged from the data themselves [18]. Each interview was coded separately in NVivo by two researchers; all coded text was compared to ensure consistency and revised after each session consistent with the constant comparative method [24]. Analysis for underlying themes was performed concurrent with coding. A narrative summary was generated to include illustrative quotes.

### Demographic and disease characteristics

To characterize our study cohort, we integrated our qualitative data with subject demographic and disease attributes obtained from a REDCap database from the previous study as well as interim data from the electronic medical record. Attributes included: age; gender; race/ethnicity; highest household education level; disease duration; and mental health status.

We determined mental health status based on the results of mental health screening during the previous study and interim events between the previous study and the interview (per subject report or medical record). A mental health history was defined as the presence of positive screen for depression or anxiety symptoms during the previous study and/or interim mental health referral and/or treatment. In the previous study, the Patient Health Questionnaire-9 (PHQ-9), a validated 9-item self-administered module [25], was used to screen for depression; the Screen for Childhood Anxiety Related Disorders (SCARED), a validated self-administered 41-item module [26], was used to screen for anxiety symptoms. A positive depression screen was defined as a score ≥5 on the PHQ-9 and included scores in the mild (5–9), moderate (10–14), moderately severe (15–19) and severe (20–27) ranges [27]. We included scores in the mild range because persistent symptoms, regardless of severity, convey an increased risk for later development of major depression and suicidal behavior [28–30]. A score of ≥1 on item 9 of the PHQ-9 questionnaire was considered indicative of suicidal ideation and also considered a positive depression screen regardless of total PHQ-9 score. A positive anxiety screen was defined as a score totaling ≥25 on the SCARED. Depression and anxiety screening was performed during outpatient rheumatology visits using REDCap (Research Electronic Data Capture) electronic survey and data capture tools hosted at CHOP [31]. Upon identification of depression or anxiety symptoms, an educational handout was provided to the family with mental healthcare referral information. Identified suicide risk was addressed with a suicide prevention protocol consisting of immediate direct questioning of suicidal intent, plan or attempt within the prior week; endorsement of any of these prompted development of a safety plan and urgent referral for immediate psychology/psychiatry evaluation.

We assessed the similarity of demographic and disease characteristics between the 16 participating and 34 non-participating dyads from the previous study. Chi-square tests were used for categorical data and Mann–Whitney tests were used for continuous data. Participating dyads were similar to the non-participating dyads, except for significantly shorter disease duration.

### Ethics, consent and permissions

Informed parental consent and child assent for study participation and publication of individual data was obtained prior to all interviews. The study was approved by the CHOP Institutional Review Board.

## Results

For the 16 youth-parent pairs, the follow-up time between participation in the previous study and interviews ranged between 1.5 and 2.5 years (mean = 2.1 years, SD = 0.3). Subject characteristics are presented in Table 1. The results for perceptions of mental health and screening and barriers/facilitators to mental healthcare are summarized below, with illustrative quotes in Tables 2 and 3, respectively. Youth with a history of mental health symptoms increased from 6 (38 %) at initial screening to 9 (56 %) at follow-up interview; the symptom and treatment status of these youth are summarized in Table 4.

**Table 1:** Demographic characteristics of participating youth with SLE/MCTD and non-participants

	Participants	Non-participants	*p*-value
	*N* = 16	*N* = 34	
Age of youth in years, mean (SD)			
At initial survey	15.0 (3.4)	15.8 (2.8)	0.25
At interview	17.0 (3.6)	–	–
Female gender of youth, N (%)	13 (81)	30 (88)	0.51
Female gender of parent, N (%)	11 (69)	–	–
Race/ethnicity of youth^a^, N (%)			0.60
White	10 (63)	14 (41)	
Black	4 (25)	14 (41)	
Asian	1 (6)	2 (6)	
Hispanic/Latino	1 (6)	2 (6)	
Other	0 (0)	2 (6)	
Highest household education level^b^, N (%)			0.14
Less than college	1 (6)	8 (24)	
College and above	15 (94)	26 (76)	
Disease duration of youth in years, median (IQR)			
At initial survey	0.9 (0.3, 2.9)	2.9 (1.3, 4.8)	0.01
At interview	3.0 (2.4, 5.0)	–	–
Initial positive mental health screen in youth^b^, N (%)	6 (38)	11 (32)	0.72
Depression	4 (25)	6 (18)	0.54
Suicidal ideation	4 (25)	3 (9)	0.12
Anxiety	5 (31)	6 (18)	0.28
Years between initial survey and interview, mean (SD)	2.1 (0.3)	–	–
Presence of mental health history^c^ in youth at interview, N (%)	9 (56)	–	–
Depression	6 (38)	–	–
Suicidal ideation	5 (31)	–	–
Anxiety	8 (50)	–	–

^a^Race/ethnicity was categorized into as the mutually exclusive groups; other includes American Indian/Alaska Native, Native Hawaiian/Pacific Islander and other)

^b^Data for these variables was obtained at time of the previous study. Highest household education level was categorized as either 1) less than college (includes incomplete college or less) or 2) college (includes completed associate, bachelors or advanced degree) and above. A positive depression screen was defined as a score ≥5 on the PHQ-9. A score of ≥1 on item 9 of the PHQ-9 questionnaire was considered indicative of suicidal ideation and also considered a positive depression screen regardless of total PHQ-9 score. A positive anxiety screen was defined as a score totaling ≥25 on the SCARED

^c^A mental health history was defined as the presence of depression or anxiety symptoms at the time of screening for the previous study and/ or mental health referral and/or treatment (per subject report or medical record) in the interim between the previous study and the interview

**Table 2:** Illustrative quotes for perceptions of mental health & screening for youth with SLE/MCTD

Mental health
*“When you feel good in your mind and you have confidence and you’re feeling able to do things, and then you can physically do it, it just makes you a happier… Mind, body, soul kind of thing…it all needs to jive. You could have a great body image, but maybe you’re depressed about something else in your life and that kind of makes your body not feel so great and then that’s all very connected.” Parent*
*“So having a good social life, that’s pretty important to be healthy. And even though maybe I’m not physically–like physically the best, like having good social and mental could make up for that…” 16 y/o male*
*“I think your mind has a lot to do with it…having a lot of your, so to speak, mental health taken care of, actually can help improve your physical health, as well. It’s equally important.” 19 y/o female*
*“Essentially with lupus, a lot of the–maybe even the original time, the lupus attack and the damage done to my kidneys was based on stress possibly. So that’s a big part of making sure I keep healthy and not being stressed out or letting things hang over my head.” 18 y/o male*
Screening in the Rheumatology Setting
*“It was helpful for me to realize, because sometimes I’ll think, oh I’m just feeling up or down, like happy or sad. But really asking, do you feel scared, do you feel anxious, do you feel like this kind of sad, that kind of sad, that was interesting. And that sort of made me think about it a little be more.” 18 y/o female*
*“It was kind of almost reassuring knowing I could answer all those questions without having to say I’m stressed out or anything like that, and that everything was going good and the satisfaction with what was going on.” 18 y/o male*
*“They were easy questions, pretty flat out, but I don’t know if I exactly told the truth, but I don’t know.” 14 y/o female*
*“I feel like it was personalized, but not in a bad way, the kind of way that it was like, do they see something in me that someone else isn’t catching, or is it–like is it written across my face that I’m worried. I didn’t feel attacked. I just felt like I wasn’t doing as good of a job as I thought I was at hiding it.” 19 year-old female*

**Table 3:** Illustrative quotes for barriers & facilitators to mental healthcare for youth with SLE/MCTD

Barriers
1) Stigma
*“You see it a lot today with mental health being a bit of a taboo. It’s not really something that people like to talk about… because you think it’ll go away or you’re exaggerating. So it’s not really an easy topic.” 19 y/o female*
*“I don’t want to disclose who am I when I want to take counseling.” Parent*
2) Fear
*“I don’t think I was fully honest, because I was so anxious and nervous…I think it was just my nerves and not wanting to talk about it because it was so scary.” 19 y/o female*
3) Uncertainty about getting help
*“And I think it’s probably a beneficial thing, but I wouldn’t even know where to start looking.” Parent* *“She wasn’t sharing because she didn’t know what to do. She didn’t know how to get back on target.” Parent*
4) Parental emotional burden
*“I didn’t realize until she told me … that she was actually hiding all that stuff … because I think she thought it was too much for me to handle.” Parent*
*“I think if their social liaisons were able to…provide you with some support groups…the opportunity for yourself to see a therapist or a specialist who can help you counsel your child, I think that would be most beneficial.” Parent*
5) Minimization by medical doctors
*“Well, she mainly gave me pamphlets that had sort of deep breathing exercises and while that helped somewhat, what I needed was more therapy and that’s when I started seeing a psychologist for cognitive brain therapy and that was more what I needed, as opposed to meditative exercises.” 18 y/o female*
6) Poor access to mental health professionals
*“The difficulty, really, is… the finance, the co-pays are frequent right now.” Parent*
*“It was difficult…we had to travel [far]…there weren’t any pediatric psychiatrists [nearby].” Parent*
*“It’s a little further away than I would like it to be due to gas prices” Parent*
*“We would schedule the next [appointment] based on, of course, my ability to take her because I had to go to work, and we still hadn’t had our own vehicle yet.” Parent*
Facilitators
1) Strong clinician relationship
*“I probably would not talk to the primary care just because we don’t have a relationship with him yet. It would start with the rheumatologist.” Parent*
2) Clinician initiative to discuss mental health
*“My first rheumatologist…recommended it. Because of what I have, he thought it was stress related. So I, fortunately, didn’t have to bring it up.” 16 y/o male*
*“He would say teenage girls have a lot going on…sometimes you can’t say things to mom…it helps to have that unbiased sounding board to just knock things off of.” Parent*
3) Clinician sincerity
*“It’s like you could tell that they really cared about your well-being. Sometimes you can tell if it’s more protocol than they really care, but it seemed like they genuinely cared about how you felt so that they could treat you better. And it just felt good.” 12 y/o female*
4) Normalization of mental health issues
*“Asking these things and thinking about them, and it should just be a normal thing. It shouldn’t be sort of weird or lie or–just part of routine.” Parent*
*“It’s after that first initial time, mentioning it, that it’s like not a problem, it’s an easy topic to talk about. Especially when they’re willing to help.” 18 y/o female*
*“You don’t have to be crazy or extremely naughty to have to use a therapist…it’s good to make it seem perfectly acceptable and fine and normal to talk to somebody in the mental health field. Because the last thing you want is a kid getting more and more depressed and keeping it in, not being able to have somebody to talk to.” Parent*

**Table 4:** Symptom and treatment status of youth with SLE/MCTD and a mental health history (*N* = 9)

Youth	Initial screen	Interim history & treatment status at interview (follow-up time)	Illustrative quotes
11 y/o Female	Anxiety	Referred at initial study; did not seek further evaluation or treatment; symptoms resolved (2.0 years)	*“I really thought that it had to do with her health…so I figured if we got the health straightened out, the rest of it would work itself out, which it did. We didn’t really address it…. She was never referred to, like, a psychiatrist or psychologist.” Parent*
17 y/o Female	Depression Anxiety Suicidal ideation	Referred at initial study; in weekly sessions with school counselor; persistent depression & anxiety (2.2 years)	*“I have anxiety issues…I went to my counselor and then I went to my mom and they both agreed and so that’s where I went.” Youth*
			*“We found it on our own. They offered it at school and we thought it would be good for her… It was free.” Parent*
17 y/o Female	Anxiety	Referred at initial study; persistent anxiety, new depression; PCP manages psychotropic medication; receives counseling through social worker at school (2.1 years)	*“First, I was having anxiety and anxiety attacks…and I think I’d been having bouts of depression for a while and just, kind of, didn’t realize it and pushed it under the rug…when I came to terms with it, I got on medicine that’s really helping, and just being more open about it helps a lot… I also have a social worker at school that I talk to.” Youth*
			*“We went in and talked to [the pediatrician] and brought it up to her, and that’s when she put her on medication…definitely something that she needed…she talks to the social worker at school, and I’ve always asked her, do you need to talk to somebody else, and at this point she doesn’t feel like she needs to because we just have such a good relationship.” Parent*
16 y/o Male	Depression Suicidal ideation	Referred at initial study; did not seek further evaluation or treatment; persistent depression (1.6 years)	*“Sometimes I feel sad, like really sad …sometimes I just don’t feel comfortable talking about that, to anyone…[my rheumatologist] did recommend a psychologist, but I was like, I don’t need it. I’m not that sad.” Youth*
19 y/o Female	Depression Suicidal ideation Anxiety	In treatment at initial study; discontinued psychiatric medications and stopped seeing therapist by time of interview; persistent depression & anxiety (2.3 years)	*“I pulled myself off of my medication for my 17th birthday…I wanted to [to go back to therapy]…I just never got around to it.” Youth*
			*“Currently, I would say she’s doing better, but she does have her own anxieties …and depression because of the illness…the infertility issues, life span issues, what lupus can and can’t do to you…and we did–I did, have her with a counselor to help with dealing with this whole illness in a teenage life on top of a female teenage life with everything else that goes on” Parent*
19 y/o Female	Depression Anxiety Suicidal ideation	In treatment at initial study; on psychotropic medication managed by PCP; irregular therapist visits; persistent depression & anxiety; history of ODD &ADHD (2.5 years)	*“Well, I mean, she was seeing a psychiatrist, like I said. She didn’t want to get up and she don’t want to go. So she been seeing a psychiatrist since she was like about four or five…she hasn’t been keeping appointments. So her primary care prescribes the Abilify and the Concerta for her. She was scheduled [for therapy], but she don’t keep the appointments.” Parent*
14 y/o Female	Negative	Concealed depression and anxiety symptoms on initial screen; subsequent suicide attempt & inpatient psychiatric hospitalization; on psychotropic medication and in psychotherapy at time of interview (2.0 years)	*“At the time, my parents didn’t know what I was feeling, going through, so I may have like said feelings sad, maybe sometimes, not all of the time… it’s been like almost two years, depression, anxiety … I see a therapist. I don’t see how talking helps, but everybody else says it does, so I try to.” Youth*
			*“As she’s getting older, she’s developing different kinds of feelings about having an illness that she needs to always think about, it’s part of her now…trying to interpret her and her emotions and it’s very hard.” Parent*
18 y/o Female	Negative	New anxiety; symptoms minimized by PCP; self-referral to counselor on advice of family friend; undergoing cognitive behavioral therapy (1.9 years)	*“I have been seeing my psychologist for anxiety for the past, I think six months or so. I started seeing a psychologist for cognitive brain therapy” Youth*
			*“She’s had some anxiety this year… and she expressed that to me…fears of being in a crowd, somebody could do harm, fears of being alone, fears that something could happen to me, if I don’t answer the text. So we did find a psychologist, a cognitive therapist” Parent*
18 y/o Female	Negative	New anxiety; referred to mental professional by rheumatologist; did not seek further evaluation or treatment (2.0 years)	*“I get anxiety and I get stressed a lot. I’m a worrier. So that’s it, it affects my health.” Youth*
			*“Emotionally, she’s a strong kid…she gets a little depressed… sometimes she can be a worrier. Every now and then, she’ll dip down and feel a little down, but she usually comes back to the top. It hasn’t bothered her to the point …where she’s sleeping all the time, but you can tell when she’s not feeling well” Parent*

### Perceptions of mental health & screening in the rheumatology setting

Almost all youth and parents placed equal importance on emotional and physical health. Many described an interaction and reliance between these two (Table 2). Some participants also noted a link between a stressed emotional state and subsequent lupus flare. Most youth reacted positively to being asked about emotions on the electronic PHQ-9 and SCARED screening questionnaires in the previous study (Table 2). Most felt at ease with the content of the questions, the electronic mode and administration in the rheumatology setting. Some felt that the screening questions helped them to become more aware of their emotions. A few reported feeling valued as a patient: “I feel that I’m not really always comfortable with telling random people how I feel, how my day went and everything. But it makes me feel like I’m important.” (17 year-old female). Another youth reported positive reinforcement of her healthy emotional state. Despite these positive reactions, some youth felt awkward, uncomfortable and vulnerable with being asked about their mental health. A few adolescents expressed a desire to conceal their true emotions. Two admitted to not answering the questions honestly.

Reactions of parents to assessment of their child’s emotional status in the rheumatology setting were also mostly positive. Eleven (69 %) said that the survey was “on target” or “was fine”, while the rest of the parents had no specific comments. All parents thought that this information was appropriate for their rheumatologist to know and thought it to be in line with their previous experience completing similar items on intake questionnaires at the PCP and rheumatology clinics. Some perceived that the mere asking of the questions indicated that their physicians cared more about their child, increased their awareness of their child’s emotions, and made them feel more comfortable to mention any emotional concerns. A few parents were grateful, stating that they had never been asked about their child’s emotions. One parent urged doctors to ask about mental health saying, “Just ask. Don’t be afraid to ask. Don’t be shy even if things look like it’s okay.” Some concerns, however, were raised about potentially overwhelming their child if the timing of the assessment was too close to diagnosis.

### Barriers and facilitators for discussing mental health concerns

We found several barriers and facilitators for youth in presenting their mental health concerns (Table 3). Youth most commonly indicated a parent as the first person that they would communicate with about emotions such as sadness and worry, followed by friends and other relatives. Parents also identified themselves as the natural initial contact for their child’s expression of stress, sadness or worry. Some described a pro-active approach by regularly checking in about their child’s feelings. Others, however, expressed feeling ill-equipped and unable to help with their child’s observed emotions due to their own emotional burden and lack of knowing how to approach the issue.

Almost all parents said that they would feel comfortable bringing concerns about their child’s mental health to the rheumatologist, whom they viewed as the primary doctor for their children. Reasons for this included awareness of an association of mental health problems with lupus, trust and confidence in the therapeutic relationship. Most parents also felt comfortable discussing concerns with the PCP for the latter reason; those who felt uncomfortable indicated a lack of established and trusted relationship with the PCP usually due to lack of continuity. One parent reported discussing mental health with another subspecialist who was viewed as the primary doctor.

In contrast, few youth said they would take the initiative to mention their feelings to their doctors. Of those who did, all said they would talk to their rheumatologist, and fewer to their PCP. Reasons for youth not mentioning emotions to doctors included: discomfort with talking about their emotions, worry about what their parents and others would think, uncertainty about what would happen, lack of awareness that emotions could be related to SLE/MCTD, and perceived disinterest of the doctor. Thus having an established relationship and accepting environment seemed to be a prerequisite for feeling comfortable enough to discuss mental health concerns. Adolescents with experience talking to doctors about their emotions were appreciative of having an opportunity to discuss their emotions and stress. They reported feeling validated and secure after talking to their physicians. Learning that sadness and worry were commonly experienced by other kids with SLE/MCTD helped to “normalize” their emotional experience: “When I talk to my rheumatologist…even if it’s not medical at all, he says…this could be causing stress, which has been really helpful to know that it’s a normal thing.” (18 y/o female) Some wished to be able to discuss emotional concerns with their doctors in between visits. Others reported that their concerns were minimized by doctors, with lack of exploration and recognition of symptom severity, and recommendation for ineffective intervention.

### Barriers and facilitators for mental health treatment

We identified barriers and facilitators (Table 3) to treatment for the nine youth with a mental health history (Table 4). Of the six youth screening positive and referred for mental health evaluation in the initial study, two were already receiving mental health treatment, two subsequently obtained treatment by the time of interview, and two did not seek treatment. Of the three youth with a newly identified mental health problem in the interim, one had an inpatient psychiatric hospitalization, one was referred but not receiving treatment, and one sought treatment on her own. Almost all parents of youth with symptoms acknowledged the importance of care by a mental health professional for their children, but they stated that initially knowing where to get help was difficult, and were appreciative of efforts made by their doctors. One symptomatic youth and her mother described delay in mental healthcare due to minimization of their symptoms by the PCP, resulting in self-referral to a psychologist on advice from a family friend.

About half of youth with a mental health history had uncertainty about whether their symptoms warranted pursuing mental healthcare, often expressing fear and shame about their emotions, stigmatized by their feelings and hoping that they would just resolve with time. Of the youth that did not seek treatment, the most common reason both youth and their parents gave was that the symptoms didn’t seem severe enough, despite a mental health referral by their rheumatologist. Yet five of the six youth (83 %) initially screening positive had persistent or additional symptoms by the time of interview. One youth, who admittedly concealed her emotional distress at the time of initial screening, had subsequent inpatient psychiatric hospitalization for suicidal attempt as her first mental healthcare.

Symptomatic youth receiving mental health treatment increased from 2 out of 6 (33 %) at initial screening to 6 out of 9 (67 %) at follow-up interview, over a follow-up time range of 1.6 to 2.5 years. Participants in the care of psychologists and psychiatrists described challenges in accessing mental healthcare due to limited availability of mental health professionals, insurance coverage restricting choice of clinicians, high copays, and inconvenience and costs of travel. However, these initial barriers were usually overcome, and parents were willing to accept the inconvenience and costs due to the necessity for services. Several other youth were taking psychotropic medications managed by their PCP, and were receiving counseling through their school, which was viewed as convenient and affordable. Several parents described wanting to further help their children themselves, but felt ill-equipped due to lack of knowledge about mental health and their own emotional burden.

## Discussion

Depression, anxiety and other mental disorders are prevalent in pediatric SLE/MCTD [2–4], and may lead to adverse clinical and psychosocial outcomes [10–12], but these conditions are under-recognized and under-treated in affected youth [5]. Our qualitative study provides an in-depth analysis of mental healthcare for youth with SLE/MCTD from the patient and parent perspective, adding to the sparse literature in this area. Our results suggest that youth with SLE/MCTD and their parents perceive mental health as an essential component of overall health, and that pediatric rheumatology is an acceptable and preferred setting for mental health screening and referral to treatment. We identify several barriers and facilitators for youth and families for identification and treatment of mental health problems, providing insight to guide interventions that may improve mental healthcare for affected youth with SLE/MCTD. Despite limited external validity of our results, derived from a small atypical sample of youth (English-speaking, highly educated and mostly White) compared to the larger pediatric SLE/MCTD population, our sample size was adequate to achieve thematic saturation of pertinent qualitative themes (the point at which no new themes are emerging) [32] as a starting point to understanding the youth and parent perspective on mental healthcare.

Our findings support cited factors associated with unmet mental healthcare needs such as stigma and those related to access to care [33], but we also identify previously unreported factors that pertain more specifically to youth with SLE/MCTD in subspecialty care. Although recent efforts have focused on augmenting the role of PCPs in delivering mental healthcare to adolescents [17], most of our participants viewed rheumatologists as their primary doctors, identifying them as a preferred first healthcare contact for mental health issues. This was attributed to the strong relationship of youth and parents with rheumatologists due to frequent visits and their reliance on them to interpret symptoms in the context of a highly complex disease. These findings suggest that, from the perspective of families, rheumatologists have a significant opportunity to facilitate their mental health diagnosis and referral.

Most of our sample of youth and parents wanted rheumatologists to ask about mental health, viewing it as a sign of optimal and comprehensive patient care. Yet, the fact that several youth were reluctant to discuss their emotions due to fear and shame points to the potential benefit for medical doctors to “normalize” mental health evaluation by making it a regular part of care. One way to achieve this is to administer mental health screening questionnaires to all youth with SLE/MCTD. In this study we found that screening youth with SLE/MCTD using electronic administration of validated screening tools in the outpatient pediatric rheumatology setting was acceptable to youth and parents. The screening process increased youth and parents’ willingness to discuss mental health issues, a benefit reported for mental health screening in other settings [34]. Screening also raised the awareness of potential mental health problems in SLE/MCTD, whether due to central nervous system disease or psychosocial distress. Although a few parents raised concerns about mental health evaluation being overwhelming too soon after diagnosis, most desired more education about potential mental health issues early in the disease process. These findings suggest that adolescents with SLE/MCTD may benefit from routine mental health screening in pediatric rheumatology, and that clinicians should be prepared to provide mental health resources to support youth and their families from the time of diagnosis.

Screening in itself, however, did not appear to be enough to de-stigmatize mental health issues. There emerged a clear value of in-person mental health assessment to create an environment conducive to discussing mental health issues, putting the screening results into context and identifying hidden feelings of self-harm. The facilitating factors that we identified suggest that pediatric rheumatologists can best create this environment by taking the initiative to begin these conversations, expressing sincere interest in mental well-being, taking mental health concerns seriously and facilitating the initial connection with mental health professionals. Additionally obtaining a family mental health history may help normalize mental health for patients and families as well as assess risk for youth; we did not address this possibility, but future studies could explore this potential effect. Given the high prevalence of suicidal ideation in our small sample and other cohorts of youth with SLE [5, 35], pediatric rheumatologists may have an opportunity to help youth avoid self-harm and disruptive inpatient psychiatric stays. Rheumatologists, however, may need additional mental health training and resources to effect an environment in which mental health is a usual part of care. Implementation of routine mental health assessment requires training of staff and integration into the clinician workflow. Encouragingly, this has been achieved in other pediatric subspecialty settings such as endocrinology for type 1 diabetes patients [36].

A major strength of our study is the detailed follow-up of a sample of youth with SLE/MCTD and mental health symptoms, providing insight into the factors influencing their receipt of mental healthcare. While our purposive sampling strategy may over-represent those with psychosocial challenges, the prevalence of mental health symptoms in our qualitative sample is similar to other studies [2, 5, 37], and our study design allowed in-depth characterization of those with mental health problems who would likely be otherwise under-represented due to selection bias. We are encouraged that symptomatic youth receiving mental health treatment increased from 33 % at initial screening to 67 % at follow-up interview. We speculate that this may be due to enhanced symptom identification through formal screening in the previous study, increased engagement in addressing mental health issues among rheumatologists caring for participants, and heightened awareness of mental health among participants, leading to increased receptiveness to rheumatologists helping with mental health issues. This underscores the opportunity for rheumatologists to help improve identification of mental health problems for affected youth.

Nevertheless, several symptomatic youth experienced delays in mental health treatment due to minimization of their symptoms by themselves, their parents and clinicians, emphasizing the need for educational resources for patients, families and clinicians. Importantly, increasing awareness of youth and families about the potential for neuropsychiatric manifestations of SLE to occur throughout the disease course [38], as well as the high occurrence of co-morbid mental disorders, may facilitate youth and parent reporting of mental symptoms. Yet it should be noted that symptom minimization and other identified barriers to mental health treatment for youth with SLE/MCTD, such limited availability and high costs of mental health services, are likely similar for youth with other chronic disease in subspecialty care. This supports a general need for healthcare systems to facilitate comprehensive care of such patients by integration of medical and mental healthcare. With the growing evidence for efficacy of collaborative care models [17, 39], co-locating mental health professionals with pediatric rheumatology and other subspecialists may alleviate the current challenges to mental health treatment.

Care delivery through these models requires significant support from healthcare systems and institutions, however, which is difficult to obtain for pediatric rheumatologists who are small in number due to limited a workforce [40]. Therefore collaborative care models, although a promising strategy for mental healthcare of youth with SLE/MCTD, are not currently feasible for all pediatric rheumatology settings. While efforts should be made by pediatric rheumatologists to advocate for medical and mental healthcare integration, other options should be explored. For example, several youth receiving school-based counseling and management of psychotropic medications through their PCPs found these alternatives to be convenient and cost-effective. Improved communication and care coordination between rheumatologists, PCPs and mental health providers is likely to help ensure that treatment is available. More research is needed to determine the optimal strategies for effective and accessible mental health treatment for youth with SLE/MCTD.

## Conclusion

Youth with SLE/MCTD and their parents perceive pediatric rheumatologists as a preferred source for mental health screening, guidance and referral. Routine mental health screening and clinician-initiated discussion of mental health may facilitate identification of youth with mental disorders. Further research is needed to implement interventions that address barriers and enhance facilitators to improve mental healthcare for youth with SLE/MCTD.

## Abbreviations

CHOP: Children’s Hospital of Philadelphia
MCTD: mixed connective tissue disease
PHQ-9: patient health questionnaire-9
REDCap: research electronic data capture
SCARED: screen for childhood anxiety related disorders
SLE: systemic lupus erythematosus
